# Novel Methodology for Identifying the Occurrence of Ovulation by Estimating Core Body Temperature During Sleeping: Validity and Effectiveness Study

**DOI:** 10.2196/55834

**Published:** 2024-07-05

**Authors:** Daisuke Sato, Koyuki Ikarashi, Fumiko Nakajima, Tomomi Fujimoto

**Affiliations:** 1 Sports Physiology Laboratory Department of Health and Sports Niigata University of Health and Welfare Niigata Japan

**Keywords:** menstrual cycle, ovulation, biphasic temperature shift, estimation method, women

## Abstract

**Background:**

Body temperature is the most-used noninvasive biomarker to determine menstrual cycle and ovulation. However, issues related to its low accuracy are still under discussion.

**Objective:**

This study aimed to improve the accuracy of identifying the presence or absence of ovulation within a menstrual cycle. We investigated whether core body temperature (CBT) estimation can improve the accuracy of temperature biphasic shift discrimination in the menstrual cycle. The study consisted of 2 parts: experiment 1 assessed the validity of the CBT estimation method, while experiment 2 focused on the effectiveness of the method in discriminating biphasic temperature shifts.

**Methods:**

In experiment 1, healthy women aged between 18 and 40 years had their true CBT measured using an ingestible thermometer and their CBT estimated from skin temperature and ambient temperature measured during sleep in both the follicular and luteal phases of their menstrual cycles. This study analyzed the differences between these 2 measurements, the variations in temperature between the 2 phases, and the repeated measures correlation between the true and estimated CBT. Experiment 2 followed a similar methodology, but focused on evaluating the diagnostic accuracy of these 2 temperature measurement approaches (estimated CBT and traditional oral basal body temperature [BBT]) for identifying ovulatory cycles. This was performed using urine luteinizing hormone (LH) as the reference standard. Menstrual cycles were categorized based on the results of the LH tests, and a temperature shift was identified using a specific criterion called the “three-over-six rule.” This rule and the nested design of the study facilitated the assessment of diagnostic measures, such as sensitivity and specificity.

**Results:**

The main findings showed that CBT estimated from skin temperature and ambient temperature during sleep was consistently lower than directly measured CBT in both the follicular and luteal phases of the menstrual cycle. Despite this, the pattern of temperature variation between these phases was comparable for both the estimated and true CBT measurements, suggesting that the estimated CBT accurately reflected the cyclical variations in the true CBT. Significantly, the CBT estimation method showed higher sensitivity and specificity for detecting the occurrence of ovulation than traditional oral BBT measurements, highlighting its potential as an effective tool for reproductive health monitoring. The current method for estimating the CBT provides a practical and noninvasive method for monitoring CBT, which is essential for identifying biphasic shifts in the BBT throughout the menstrual cycle.

**Conclusions:**

This study demonstrated that the estimated CBT derived from skin temperature and ambient temperature during sleep accurately captures variations in true CBT and is more accurate in determining the presence or absence of ovulation than traditional oral BBT measurements. This method holds promise for improving reproductive health monitoring and understanding of menstrual cycle dynamics.

## Introduction

Understanding the menstrual cycle and fertility period is important for those who wish to conceive or use contraception. The fertile period starts 5 days before the day of ovulation, with a higher probability of conception on the day before ovulation [1-4]. Although several biomarkers have been used to predict the day of ovulation, measuring basal body temperature (BBT) immediately after waking is a simple and noninvasive method [5]. Indeed, a cohort study reported that 21% of women use BBT to track their fertility [6], so it is very important to improve the accuracy of BBT in predicting the presence or absence of ovulation.

The nadir, the time of the lowest BBT in a menstrual cycle, is observed 1 day before ovulation, with BBT rising approximately 2 days after the luteinizing hormone (LH) peak [7]. This biphasic shift in BBT is, therefore, an effective indicator of the presence or absence of ovulation. However, there are several limitations in identifying ovulation using the BBT method. First, because of the large diurnal variation in BBT [8], it must be measured at the same time each day. In addition, multiple missing values during a cycle make it difficult to detect biphasic temperature shifts [9]. Therefore, women need to wake up a few minutes before getting out of bed to measure their BBT [10], and it has been reported that 85% of women find the BBT method too burdensome [11]. Therefore, there is an ongoing debate regarding BBT methods that are more accurate and less burdensome for women.

Although there are several limitations to understanding ovulation induction using BBT, much attention has been paid to measuring body temperature during sleep using wearable devices. Attempts have been made to predict ovulation induction based on the skin temperature during sleep [12,13]. Interestingly, wrist skin temperature (WST) during sleep has been shown to reflect biphasic temperature shifts measured using the BBT throughout the menstrual cycle [14]. In addition, research is underway to improve accuracy by simultaneously measuring 1 or 2 physiological parameters in addition to skin temperature [13]. However, the possibility that these skin temperatures may be influenced by the ambient temperature cannot be excluded. Therefore, a simple method for measuring core body temperature (CBT) that is less sensitive to ambient temperatures is desirable.

Based on the background described above, in this study, we tested a method of estimating the CBT by measuring the skin temperature of the breast and the ambient temperature simultaneously and using an algorithm to minimize the effect of the ambient temperature. We also tested the hypothesis that capturing biphasic shifts in estimated CBT would improve the accuracy of determining the presence or absence of ovulation.

## Methods

### Ethical Considerations

This study was conducted in accordance with the Declaration of Helsinki and was approved by the Ethical Committee of Niigata University of Health and Welfare (18759-211126). Informed consent was obtained from all participants prior to their inclusion in this study. The data collected in this study were anonymized to ensure that individual participants could not be identified. All personal identifiers were removed, and data were stored securely with access limited to the research team. Measures were taken to ensure the confidentiality and privacy of the participants throughout the study. However, in case some participants dropped out during the experiment, a correspondence table of individual names and participant numbers was created and saved as a separate file. Participants in experiments 1 and 2 received cash compensation of JP ¥6000 and JP ¥3000 (about US $37 and US $18), respectively. No figures, tables, or supplementary materials in this manuscript contain identifiable information about individual participants.

### Experimental Design and Procedure

This study comprised 2 experiments. The first experiment examined the validity of the methodology for estimating CBT (experiment 1). The second experiment examined the accuracy of the estimated CBT in discriminating biphasic temperature shift by comparing it with the oral temperature measured upon waking (experiment 2).

**Figure 1:**
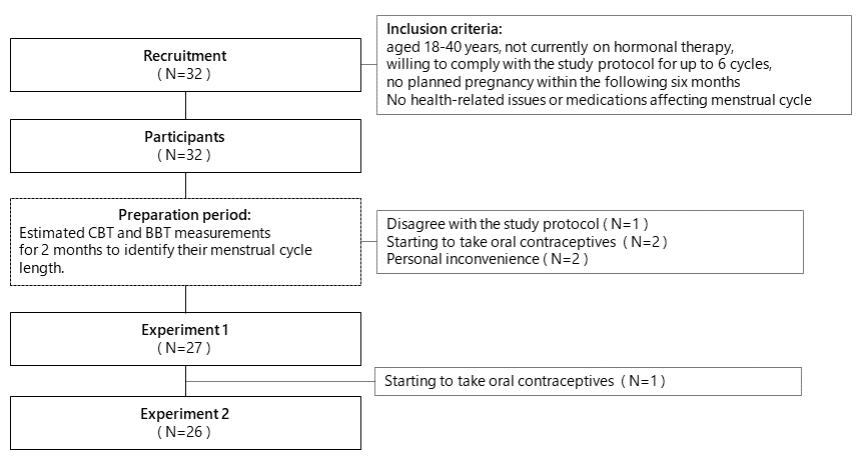
Flowchart of recruitment and participation.

Figure 1 shows the flow of recruitment and participation in this study. Participants were recruited from a university campus. The recruitment process involved contacting potential participants directly and posting on social media. Potential participants were informed about the study through email invitations. Information sessions were held to explain the purpose of the study, the procedures involved, and the expected time commitment. Through this process, 32 women who met the following inclusion criteria were recruited: aged 18 to 40 years, not currently on hormonal therapy, willing to comply with the study protocol for up to 6 cycles, and no planned pregnancy within the following 6 months [15]. The eligibility criteria included no restrictions on the regularity or length of the menstrual cycle [16].

After arranging their individual dates, the participants received a detailed briefing on the experiment in a university laboratory. Private information on age, weight, height, and time since discontinuation of hormonal contraception was collected after obtaining informed consent. All participants were instructed to wear a special night bra (Toppan Edge Inc) with a thermal sensor to bed to estimate CBT, as explained in detail in the following section, and measure BBT every morning immediately after waking using an oral thermometer (Citizen CTEB503L-E; Citizen Watch Co Ltd) 2 months before the experiment to estimate their cycle length accurately. Additionally, to estimate the presence or absence of ovulation, the participants were instructed to use an ovulation test kit (Doctor’s Choice One Step Ovulation Test Clear; Beauty and Health Research) from the day after the end of menstruation. We asked the participants to report their BBT, ovulation test results, and onset and end of menstruation using Google Forms.

After the length of each participant’s menstrual cycle was confirmed, experiment 1 was started. An ingestible sensor and wireless data recorder were used to measure CBT during sleep each day in the follicular and midluteal phases (as explained in detail in the following section), in addition to the estimated CBT and BBT described above.

After experiment 1, all participants were instructed in the laboratory on the methods to be used in experiment 2. The techniques used to determine menstrual cycle length, ovulation, and daily monitoring in experiment 1 were the same as those used in experiment 2. One participant withdrew from the study due to oral contraceptive use after completion of experiment 1.

### Estimated CBT

The estimated CBT was calculated using the heat flux method, which is a known method for estimating CBT. According to Fourier’s law, heat flux (q) is proportional to the temperature gradient (δt/δx) and thermal conductivity (λ), as illustrated in the following equation:

q=λ(δt/δx)

**Figure 2:**
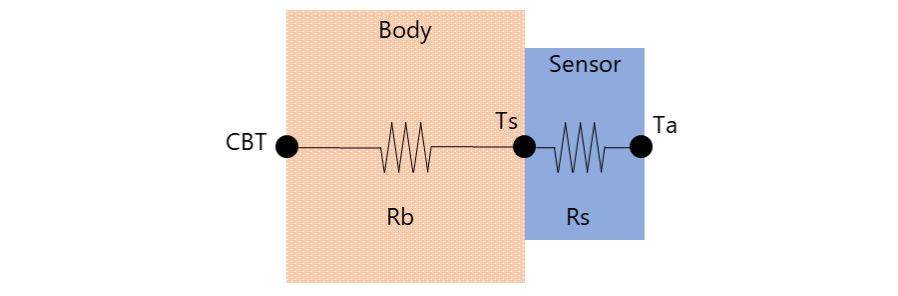
The body temperature measurement model.

Applying this to the body temperature measurement model shown in Figure 2, the CBT is adjusted to the skin temperature (*Ts*), which is influenced by the body’s resistance (*Rb*). Subsequently, skin temperature is adjusted to the ambient temperature (*Ta*) under the resistance of the sensor (*Rs*). The heat flux for both the body and sensor is represented by the following 2 equations:

q = λ1(CBT – Ts)

q = λ2(Ts – Ta)

Using these 2 equations, the CBT was calculated with the following equation:

CBT = Ts + K(Ts – Ta) ※K = λ2 / λ1

*K* represents a coefficient that includes the thermal conductivity of both the sensor and body, and it can also be derived from the values obtained through measurements.

At this time, we converted the formula for calculating the CBT as follows:

Ts = (K / (1 + K))Ta + CBT(1 + K)

Substitution of the experimentally determined skin temperature and the ambient temperature into the above equation gives the slope (α) of this linear function:

α = K(1 + K)

By converting this expression into the following, *K* can be calculated from the experimental data:

K = α/(1 – α)

As the structure of the sensor remained consistent, its thermal conductivity was considered constant. Similarly, the body’s thermal conductivity is viewed as stable because fat and muscle mass do not change significantly over a brief period for the same individual. Therefore, for the same individual, *K* can be considered a constant, whereas *K* varies for different individuals. However, the accuracy of absolute temperature values is less critical in predicting the menstrual cycle. What matters more in this verification is the daily temperature fluctuation within each person. Thus, rather than calculating the thermal conductivity for each participant, a general coefficient of thermal conductivity, *K*, was established based on the data from previous participants using the sensor.

In this method, the estimated CBT was recorded every minute while sleeping. The initial hour’s data after application of the sensor were deemed unreliable owing to unstable heat flux, so we set the measurement period to the subsequent 4 hours. To mitigate the effect of external heat, readings in which ambient temperature was higher than skin temperature were excluded from the analysis. The average temperature over these 4 hours was taken as the representative temperature. Theoretically, if the structure and individuals remain unchanged, the thermal conductivity is considered to be constant. However, shifts in thermal conductivity can occur if the sensor is moved away from the skin or if there is a rapid change in ambient temperature, leading to significant variations in the estimated CBT. Hence, any large fluctuations in the estimated CBT owing to sensor displacement or swift changes in ambient temperature were deemed anomalies and excluded from the analysis.

### True CBT

True CBT was measured using an ingestible temperature sensor and a wireless data recorder (e-Celsius; BodyCap). The participants visited the laboratory the evening before the day of the follicular and midluteal phase measurements and were instructed to swallow a pill-shaped sensor with water approximately 2 hours before bedtime. Data were sampled every minute, which allowed a maximum of 33 hours of measurement, and retrieved via Bluetooth when the participants revisited the laboratory the following morning and averaged over the sleeping period.

### LH Test

Participants underwent a home-based urine LH test using an ovulation test kit (Doctor’s Choice One-Step Ovulation Test Clear; Beauty and Health Research) for each cycle, following the manufacturer’s instructions. Home-based LH tests are prevalent tools for detecting ovulation and determining the fertile window [17]. The participants were instructed to continue with the daily LH test until they observed a positive result, signified by a distinct line on the device, or until the onset of their next menstruation. A positive result signifies an LH surge, typically preceding ovulation by 1 day [9,10]. The participants logged the test results using Google Forms. A cycle registering a positive LH test result was labelled an ovulatory cycle, whereas one showing only negative results was deemed an anovulatory cycle. The LH test was used as the benchmark to assess the diagnostic accuracy of the specified temperature methods.

### Data Analysis

Experiment 1 examined the relationship between the estimated CBT, calculated using the chest skin temperature and ambient temperature, and the true CBT, assessed using ingestible sensors. Kolmogorov-Smirnov tests and 2-tailed paired *t* tests were performed using PASW Statistics in SPSS (version 27; IBM Corp). The Kolmogorov-Smirnov test revealed normal distributions for both sets of temperature data. A 2-tailed paired *t* test was used to compare the true and estimated CBT in each menstrual phase and the between-phase temperature changes. Additionally, correlations among true CBT, estimated CBT, skin temperature, and ambient temperature at the follicular and luteal phases were examined using repeated measures correlation with the R (R Project for Statistical Computing) package *rmcorr*. The *rmcorr* correlation coefficient (rrm) determines the common intraindividual relation for paired measurements assessed on 2 or more occasions for multiple individuals [18]. The significance level was set at 5%.

Experiment 2 used the results of the LH test as a benchmark to test the accuracy of the 2 temperature measurement methods (estimated CBT and oral BBT) in determining ovulatory cycles. In this experiment, all cycles were included in the analysis, regardless of the presence or absence of ovulation as assessed by the LH test. The final analysis excluded cases with missing LH test results or missing temperature readings >30% for any device cycle. This resulted in the collection of 74 cycles of data from 26 women. A temperature shift was defined as occurring when 3 consecutive temperature measurements were at least 0.2 °C higher than the highest of the previous 6 measurements, or at least 4 of the previous 6 measurements if data were missing [5]. According to Freundl et al [19], 2 exceptions apply to this rule: if the third temperature is less than 0.2 °C higher than the preceding 6, a fourth measurement is required. This fourth temperature must exceed the 6 previous lower temperatures, although not necessarily by 0.2°C. If among these 3 higher temperatures, 1 drops to or below the average of the preceding 6, it can be disregarded, provided the third higher temperature is at least 0.2°C above this average. These exceptions were mutually exclusive. To discern multiple temperature shifts within a cycle, we focused on those occurring in the last 14 days, excluding shifts due to factors like illness [16]. A cycle was classified as monophasic if it lacked a temperature shift. The accuracy of these shifts was evaluated using an LH test as a benchmark. The diagnostic accuracy measures included sensitivity, specificity, and predictive value, considering the nested design of the study.

## Results

### Relationship Between True and Estimated CBT (Experiment 1)

Table 1 shows the true and estimated CBT in the follicular and midluteal phases and the between-phase temperature changes. There were significant differences between the true and estimated CBT in both the follicular (*P*<.001) and midluteal phases (*P*<.001). However, the difference between the 2 menstrual phases did not differ between the true and estimated CBT groups (*P*=.10).

**Table 1:** Mean true and estimated core body temperature (CBT) in follicular and midluteal phases. There was a significantly higher true CBT than estimated CBT in both follicular and midluteal phases. However, between-phase temperature change did not differ between true and estimated CBT.

Phases	True CBT (ºC), mean (SD)	Estimated CBT (ºC), mean (SD)	*P* value
Follicular	36.48 (0.22)	36.19 (0.27)	<.001
Midluteal	36.91 (0.21)	36.55 (0.24)	<.001
Between-phase temperature change	0.41 (0.20)	0.34 (0.24)	.10

**Figure 3:**
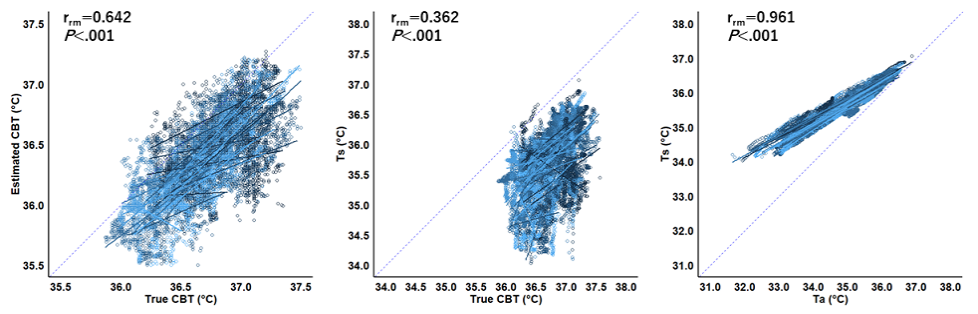
Repeated measures correlation plot for averaged data of 2 temperatures at the follicular and luteal phases.

**Figure 4:**
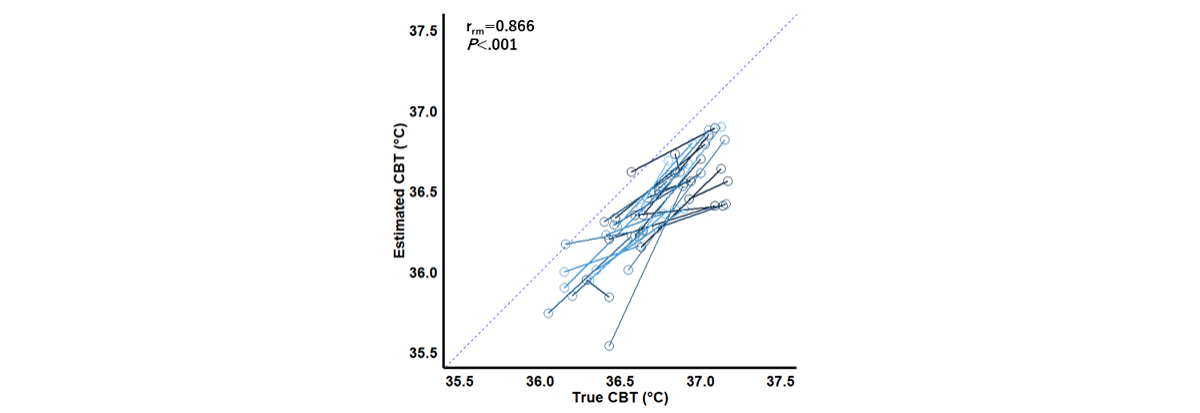
Repeated measures correlation plot for averaged data of 2 temperatures at the follicular and luteal phases.

Figure 3 shows a repeated measures correlation plot for data for each second for the 2 temperatures. Positive correlations were found between true and estimated CBT (rrm=0.642, 95% CI 0.631-0.652; *P*<.001), true CBT and skin temperature (rrm=0.362, 95% CI 0.347-0.377; *P*<.001), and ambient temperature and skin temperature (rrm=0.961, 95% CI 0.960-0.962; *P*<.001). Figure 4 shows a repeated measures correlation plot of averaged data for true and estimated CBT. We found a positive correlation between true and estimated CBT at the follicular and midluteal phases (rrm=0.866, 95% CI 0.739-0.934; *P*<.001).

### Diagnostic Accuracy of Ovulation Cycle (Experiment 2)

Table 2 shows the diagnostic accuracy of estimated CBT and BBT. The *χ*^2^ test revealed no significant difference in the percentage of the cycles with a biphasic shift in temperature between estimated CBT and BBT (*P=*.11). Using the LH test as the reference standard for ovulation, biphasic temperature shifts observed in the estimated CBT were more accurate for detecting the occurrence of ovulation in the menstrual cycle than BBT, followed by the McNemar test (*P=*.01).

**Table 2:** Diagnostic accuracy of estimated core body temperature (CBT) and basal body temperature (BBT).

Variables		Estimated CBT	BBT
Cycles with biphasic shift (n=74), n (%)		52 (70)	44 (60)
Cycles with no biphasic shift (n=74), n (%)		22 (30)	30 (40)
**Diagnostic accuracy (urine luteinizing hormone tests as standard reference; n=60)**			
	True positives, n (%)	51 (69)	41 (55)
	True negatives, n (%)	13 (18)	11 (15)
	False positives, n (%)	1 (1)	3 (4)
	False negatives, n (%)	9 (12)	19 (26)
	Sensitivity	85.00	68.33
	Specificity	92.86	78.57
	Negative predictive value	98.08	93.18
	Positive predictive value	59.09	36.67
	*F*_1_-score	0.91	0.79

There was no significant difference in the percentage of cycles with a biphasic shift in temperature between estimated CBT and BBT. On the other hand, the McNemar test revealed that the biphasic temperature shift based on estimated CBT was more accurate for detecting the occurrence of ovulation in the menstrual cycle than BBT.  

## Discussion

This study evaluated a method for estimating CBT by simultaneously monitoring skin temperature and ambient temperature during sleep. It also tested the hypothesis that detecting biphasic shifts in estimated CBT would improve the accuracy of determining the presence or absence of ovulation. The following 2 main findings were obtained: first, estimated CBT derived from skin and ambient temperature during sleep was significantly lower in both the follicular and midluteal phases compared to digestive temperatures (true CBT). However, the differences between these 2 phases were similar for the true and estimated CBT values. In addition, a significantly repeated measures correlation was observed between the true and estimated CBT. This suggests that although the estimated CBT does not directly assess the true CBT, it effectively reflects its fluctuations. Second, this method of estimating the CBT showed greater sensitivity and specificity in detecting ovulation than traditional BBT measurements taken orally at awakening. These results suggest that the estimated CBT approach is a valuable tool for reproductive health monitoring.

The results of experiment 1 indicate that the estimated CBT derived from skin temperature and ambient temperature is a reliable marker for monitoring CBT. Previous research has demonstrated the superiority of CBT over skin and oral temperatures for assessing BBT [20,21]. The accuracy of CBT measurements is critical for accurately delineating biphasic temperature shifts associated with the menstrual cycle [22,23]. Recently, there has been an increase in research attempting to understand the biphasic shifts in body temperature using skin temperature measurements. However, these measurements have the limitation of being affected by environmental conditions [24]. In this study, skin temperature was found to be significantly lower than digestive temperature (a direct indicator of true CBT) and estimated CBT and showed a strong correlation with ambient temperature. Furthermore, the estimated CBT showed a more robust correlation with the true CBT (rrm=0.642), while skin temperature maintained a significant positive association with the true CBT (rrm=0.362). Consequently, the method used in this study to estimate the CBT may provide a feasible, noninvasive approach to monitoring CBT.

In experiment 2, the estimated CBT calculated using skin temperature and ambient temperature during sleep showed greater accuracy in detecting ovulation (the occurrence of an LH surge) than the conventional method using oral temperature. This advancement is significant for the physiological monitoring of women’s health. Traditional BBT, which uses oral temperature measured immediately upon waking, requires precise timing every day. Missing data from this method can hinder the detection of biphasic temperature shifts [9]. It also requires women to wake up several minutes before getting out of bed to measure their BBT, which 85% of women find inconvenient [11]. Therefore, new methods for detecting ovulation that involve measuring the skin temperature at rest and during sleep have been validated [12,16]. In particular, measurements taken during stable sleep states eliminate fluctuations caused by daytime activities, leading to a more accurate understanding of the menstrual cycle [16]. In this study, we performed measurements during sleep to minimize the influence of variable factors on the measurement protocol. In addition, our method for calculating the estimated CBT includes ambient temperature in the equation to adjust for external environmental effects, potentially improving the accuracy of biphasic detection.

The results of this study suggest that CBT can be estimated using skin temperature and ambient temperature. Daily monitoring of CBT is crucial for detecting biphasic shifts in BBT that occur during the menstrual cycle. However, traditional methods for measuring CBT involve measuring esophageal or rectal temperatures, which are invasive. The noninvasive technique for estimating CBT developed in this study is particularly useful for identifying biphasic shifts in BBT because it avoids the difficulties associated with regular invasive measurements. In addition, recording temperature during sleep helps reduce the variability caused by physical activity, resulting in more consistent readings [14]. Moreover, the ability of this estimation method to account for the effects of ambient temperature is particularly important for understanding menstrual cycles in regions with extreme weather conditions or where it is difficult to control the ambient temperature.

The prediction of ovulation via BBT is vulnerable to disruption from a multitude of factors, including fever, alcohol consumption, emotional or physical stress, sleep disturbances, variations in room temperature, changes in physical activity, climatic fluctuations, and the recent initiation or cessation of contraceptive pills or antipyretics [5]. Despite these susceptibilities, BBT is widely used for both contraceptive purposes and the assessment of ovulatory function, as the estimated BBT provides a noninvasive, straightforward metric that consistently delineates ovulation through discernible shifts during the menstrual cycle. Thus, these findings offer valuable practical insights, yet they must be approached with prudence. Furthermore, a significant limitation of BBT is its inability to forecast ovulation proactively, thereby rendering it ineffective for pinpointing the fertile window. Additionally, this research used the detection of the LH surge to ascertain ovulation. More precise methodologies, such as ultrasonography or the analysis of reproductive hormone concentrations in serum and saliva, are required. It is also important to note that all the study participants were Japanese. Future studies should include a more diverse range of physiological and ethnic backgrounds to confirm the wider applicability of these findings.

This study demonstrated that the estimated CBT derived from skin and ambient temperatures during sleep accurately captured variations in the actual CBT. Furthermore, this estimation method has proven to be more precise in determining the occurrence or absence of ovulation than traditional methods, which rely on oral temperature measurements taken immediately upon waking.

## Abbreviations

BBT: basal body temperature
CBT: core body temperature
LH: luteinizing hormone
WST: wrist skin temperature

## Data Availability

The data are available from the corresponding author on reasonable request.
